# Influence of Parasitic Capacitance on Output Voltage for Series-Connected Thin-Film Piezoelectric Devices

**DOI:** 10.3390/s121216673

**Published:** 2012-12-04

**Authors:** Kensuke Kanda, Takashi Saito, Yuki Iga, Kohei Higuchi, Kazusuke Maenaka

**Affiliations:** 1 Graduate School of Engineering, University of Hyogo, 2167 Shosha, Himeji 671-2280, Japan; E-Mail: maenaka@eng.u-hyogo.ac.jp; 2 Japan Science and Technology Agency, 8111 Open Labs, 2167 Shosha, Himeji 671-2280, Japan; E-Mails: t-saito@eratokm.jp (T.S.); iga@eratokm.jp (Y.I.); k-higuchi@eratokm.jp (K.H.)

**Keywords:** output-voltage enhancement, SPICE, parasitic capacitance, piezoelectric thin films, MEMS

## Abstract

Series-connected thin film piezoelectric elements can generate large output voltages. The output voltage ideally is proportional to the number of connections. However, parasitic capacitances formed by the insulation layers and derived from peripheral circuitry degrade the output voltage. Conventional circuit models are not suitable for predicting the influence of the parasitic capacitance. Therefore we proposed the simplest model of piezoelectric elements to perform simulation program with integrated circuit emphasis (SPICE) circuit simulations). The effects of the parasitic capacitances on the thin-film Pb(Zr, Ti)O_3_, (PZT) elements connected in series on a SiO_2_ insulator are demonstrated. The results reveal the negative effect on the output voltage caused by the parasitic capacitances of the insulation layers. The design guidelines for the devices using series-connected piezoelectric elements are explained.

## 1. Introduction

For several decades, there has been increasing interest in the healthcare industry for human healthcare monitoring systems. This has led to an increase in research on this topic. The medical and welfare costs to governments worldwide have now reached critical levels. Various human-activity monitoring systems have been developed and are commercially available. To realize such monitoring systems, miniaturization and integration of sensors are essential. A small, robust, integrated, wireless, and low-power-consumption sensor system would have a significant impact on patient monitoring in the human-healthcare and wellness industries. Various healthcare monitoring systems using micro-electro-mechanical-systems (MEMS) sensors have been reported [1,2]. Piezoelectric materials have attracted attention for the application of miniature and low-power-consumption sensors [3,4]. Piezoelectric materials are suitable for MEMS devices with low-power-consumption because they can generate output voltages without the need for a capacitance-to-voltage converter as in electrostatic-type sensors, or the need to apply an electrical current to piezo-resistive sensors. Various MEMS sensors have been developed using piezoelectric materials, such as ultrasonic transducers [5], accelerometers [6,7], gyroscopes [8], force sensors [9], pressure sensors [10], and gas sensors [11]. The sensing principles for the physical quantities include charge, voltage, and resonant-frequency measurement. In general, the output signal decreases with the characteristic scale of the sensor device. Therefore, peripheral circuitries for signal amplification, filtering, and operation are necessary for such devices that output small signals. For sensors to be incorporated into a non-invasive monitoring system that can operate automatically for a long period of time, an overall reduction in power consumption is required. Also, since the circuits consume energy, the circuit size should be as small as possible.

**Figure 1.**
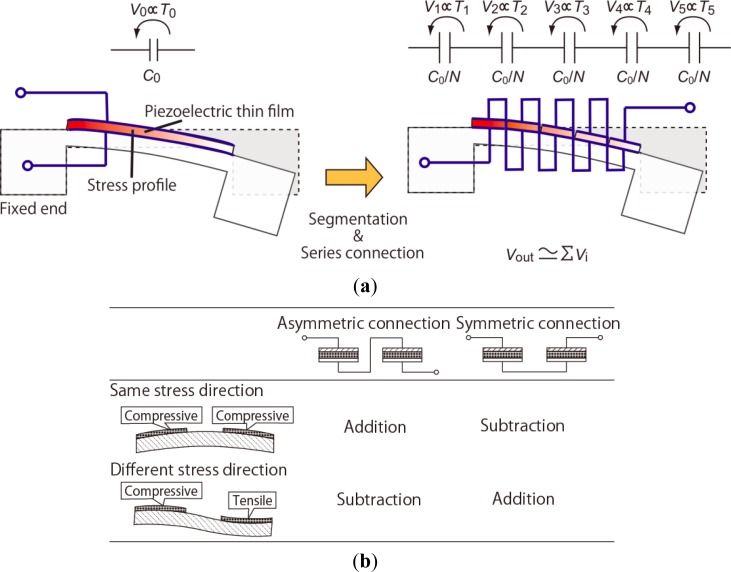
Diagram of output-voltage operation using series-connected piezoelectric elements. (**a**) Diagram of output multiplication effect using series-connected piezoelectric elements; (**b**) The output voltage is increased or decreased on the basis of the method of connection and the directions of stress.

**Figure 2.**
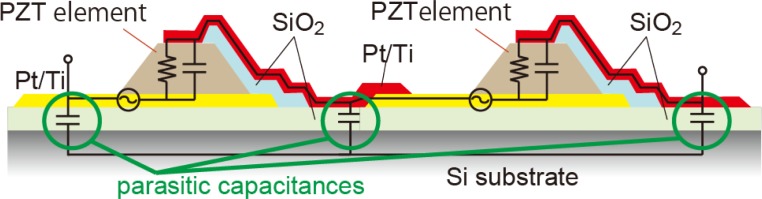
Insulation layers below bottom electrodes form parasitic capacitances.

Previously, we have reported fine-resolution processing techniques for piezoelectric Pb[Zr,Ti]O_3_ (PZT) thin films [12] and proposed a procedure for signal operation for a MEMS mechanical structure using series-connected segmented PZT elements [13,14]. A schematic illustration of the device operation is shown in Figure 1(a). The piezoelectric films are sandwiched between the top and bottom electrodes and fabricated on the mechanical structures such as cantilevers and membranes. Electric charges are generated on the electrodes when the structure deforms and are proportional to the stress in the PZT thin films. On the piezoelectric thin film, the in-plane stress is dominant because of the bending mode of the structure deformation. If a piezoelectric thin film is segmented into *n* elements with equal areas which are electrically connected in series, the combined capacitance of these elements becomes 1/*n* of the original thin film, while the net output charge remains constant. This results in an output voltage that is *n* times greater than that of the original film. Here, the in-plane stress is assumed to be uniform over the film, and the area loss due to the fill factor of piezoelectric elements is assumed to be negligible. In this study, the enhancement effect on the output voltage is referred to as the multiplication. The output voltage of series-connected elements is canceled when two elements experience stresses with different signs (*i.e.*, tensile and compressive stresses), as shown in Figure 1(b). We have experimentally verified the output-voltage multiplication and cancelation [14,15]. However, output voltages reach a limit as an increasing number of elements are connected, resulting from the parasitic capacitance formed by the insulation layer underneath the bottom electrodes, as shown in Figure 2.

Output-voltage multiplication has significant potential in the development of energy harvesters with high output voltages, which are greater than the built-in voltage of the p-n junction, and sensors with high signal-to-noise ratios. Ideally, the output voltage would increase to infinity with the increasing number of elements. However, the parasitic components in the measurement system prevent this from happening. In the design of devices that employ output-voltage multiplication, estimation of the optimum number of series connections and the effect of parasitic capacitances are important. However, the system becomes very complicated when the number of connections increases, and therefore the estimation of the parasitic capacitance becomes complicated. This study proposes a simple procedure using simulation program with integrated circuit emphasis (SPICE) to estimate the effect of parasitic capacitances and specifies a design guideline for series-connected piezoelectric elements.

## 2. Circuit Models of Series-Connected Piezoelectric Elements with Parasitic Capacitances

### 2.1. Equivalent Circuit Models of Piezoelectric Elements

For the thin-film piezoelectric elements, the mechanical behavior, including the stress in the piezoelectric film, is determined by the substrate deformation. This is because the film has negligible thickness compared to that of the substrate structure. For piezoelectric bulk transducers, some equivalent circuits have been proposed. Mason’s models, which are derived from the constituent equation of piezoelectric materials, are generally used as equivalent circuits for piezoelectric transducers [16].

In this paper, we investigate the influence of parasitic capacitances on the output voltage generated from series-connected piezoelectric elements. To understand the charges obtained from the output pads of the device with taking account of the influence of the parasitic capacitance, a model with a charge source, a capacitance of the piezoelectric elements, and parasitic capacitances is sufficient. The model is based on assuming ideal piezoelectric elements without leakage, and does not take dynamic phenomena into account.

**Figure 3.**
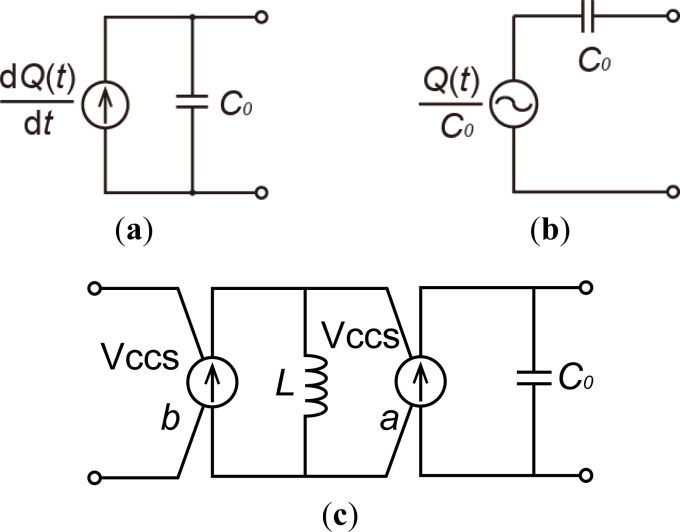
Circuit models for a charge source with a piezoelectric capacitor. (**a**) Current-source model; (**b**) Voltage-source model; (**c**) Vccs model.

The charge source is not included in the standard SPICE library. Therefore, a charge source with piezoelectric capacitance should be produced from basic SPICE elements. The models in Figure 3 show the charge sources. The model of a current source with a current of d*Q*(*t*)/d*t* connected in parallel with a piezoelectric capacitor with capacitance *C*_0_ is shown in Figure 3(a), where *Q*(*t*) is the generated electric charge. The model of a voltage source with a voltage of *Q*(*t*)/*C*_0_ connected in series with a capacitor is shown in Figure 3(b). The voltage-source model illustrated in Figure 3(b) cannot indicate when parasitic capacitances decrease the charge supplied by piezoelectric elements, *i.e.*, the voltage source fully charges all capacitors. The output charge should vary with the electrical load when the charge source is employed.

A charge source model is shown in Figure 3(c). The model is composed of two voltage-controlled current sources (Vccs), an inductor *L*, and the piezoelectric capacitance *C*_0_. The Vccs, which supplies electrical current proportional to the input voltage, is a standard element in SPICE. The constant scale factor can be set arbitrarily. The application of input voltage, *v*_i_(*t*), to the first Vccs with thescale factor *a*, produces current *a*·*v*_i_(*t*). The input voltage applied to the second Vccs with scale factor *b*, becomes the derivative value (*L*·d(*v*_i_(*t*)·*a*)/d*t*) of the current because of the inductor. Therefore, the current supplied by the second Vccs becomes *b*·*L*·d(*v*_i_(*t*)·*a*)/d*t*. If *L*, *a*, and *b* are set to 1, the current becomes d*v*_i_(*t*)/d*t*, which is the derivative of the input voltage. The input voltage should correspond to the intended charge, *i.e.*, 1 V per 1 C. The current becomes the derivative of the charge, resulting in the definition of electrical current. Therefore the Vccs model can work as a charge source model for a piezoelectric capacitor.

**Figure 4.**
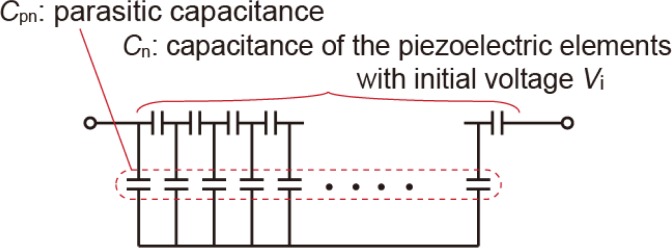
Equivalent circuit model of the series-connected piezoelectric elements. The piezoelectric elements are modeled as simple capacitors with initial voltages.

The simplest basic model proposed in this paper is a capacitor with initial voltages. Figure 4 shows the model circuit of series-connected piezoelectric elements in which parasitic capacitances are connected to the piezoelectric elements in parallel. Moreover, parasitic capacitances are connected to the electrically floating substrate. Electrical charges generated in the piezoelectric elements are defined as the initial voltages, and are distributed to parasitic capacitances. However, these parasitic capacitances partially discharge the charges on the piezoelectric elements. The effect of the parasitic capacitance is evaluated seen from the potential at the output node after charge distribution.

### 2.2. Analytical Procedure for Evaluating the Influence of the Parasitic Capacitance

The influence of parasitic capacitances on the output signal can also be estimated analytically without using SPICE. The piezoelectric film is segmented into *n* elements. Suppose that in Figure 4, the leftmost node is grounded. At the initial condition, the capacitance of the *i*-th piezoelectric element, *C*_i_, has an initial voltage *v*_i_ with an electric charge *Q*_i_ = *C*_i_*v*_i_, and the *i*-th parasitic capacitance, *C*_pi_, has no initial voltage. After charge distribution, piezoelectric elements and parasitic capacitances have electric charges, *Q*_i_’, and *Q*_pi_’, respectively. The electrical potentials at the right-side node of each piezoelectric element and at the substrate are *V*_i_ and *V*_s_, respectively. The nodal potentials are calculated from the law of charge conservation. The relationship between the initial and equilibrium charges is expressed by the following simultaneous matrix equation:

(1) {AQ'+IQp'=AQCQ'+CpQp'=0

where **A**, **C**, and **C_p_** are *n* × *n* matrices. *A*_jk_ is −1 for *j* = *k*, 1 for *j* = *k* + 1, and 0 otherwise. *C*_jk_ is 1/*C*_i_ for *j* = *k*, except for *k* = *n*, and *C*_jk_ is 0 for *j* ≠ *k* or for *j* = *n. C*_pjk_ is 1/C_pi_ for *j* = *k* except for *k* = *n*, −1/*C*_pi_ + 1 for *k* = *j* + 1, −1 for *j* = *n*, and 0 otherwise. **Q’** = {*Q*_1_’, *Q*_2_’ … *Q*_n_’}, **Q_p_**’ = {*Q*_p1_’, *Q*_p2_’ … *Q*_pn_’}, **Q** = {*Q*_1_, *Q*_2_ … *Q*_n_}, and **I** is an *n* × *n* identity matrix. From the equation, the electric charges in the piezoelectric elements, **Q’**, are expressed as follows:

(2) $$Q'={\left(I+GC_{p}{}^{-1}C\right)}^{-1}$$

where **G** is a unit lower triangular matrix. The resulting output potential, *V*_n_, is the summation of (*Q*_i_’/*C*_i_). The influence of the parasitic capacitance is evaluated as a multiplication factor, which is defined as the ratio between the obtained output voltage, *V*_n_ and the initial voltage from the thin film before segmentation. When the thin film is segmented into elements with equal areas (each element has capacitance, *C*, and parasitic capacitance, *C*_p_), the equation can be expressed as a ratio of the parasitic capacitance to piezoelectric capacitances, *C*_p_/*C*. Therefore, the relation between the charges, *Q* and *Q’*, is defined by the ratio *C*_p_/*C*, and is not related to the absolute value of *C* or *C*_p_. Although the analytical solution can be obtained from the matrix formula, it becomes too complex when the segmented number is large.

The use of the circuit simulation tool, SPICE, for the estimation is much easier than the analytical procedure. SPICE transient analysis of the circuit model of capacitors with initial voltages can demonstrate the charge distribution after reaching an equilibrium state. Although the charge source is not prepared in SPICE, the previously mentioned model of the capacitor with an initial voltage makes it possible to use SPICE to easily estimate the influence of the parasitic capacitances. The transient analysis in the SPICE software has the option of providing initial voltages to capacitors. The equilibrium solution of the transient analysis provides nodal potentials after charge distribution. The influence of the parasitic capacitance is evaluated as the multiplication factor.

## 3. Influence of the Parasitic Capacitances

To evaluate the influence of the parasitic capacitances, the output voltage from series-connected piezoelectric elements is calculated using SPICE. The capacitance of the piezoelectric thin film is given by *C* = *ε*_0_·*ε*_piezo_·*S*/*t*_piezo_, where *ε*_0_ is the dielectric constant of a vacuum, *ε*_piezo_ is the relative dielectric constant of the piezoelectric material, *S* is the area of the electrode, and *t*_piezo_ is the thickness of the piezoelectric thin film. The parasitic capacitance is given by *C*_p_ = *ε*_0_· *ε*_ins_·*S*/*t*_ins_, where *ε*_ins_ is the relative dielectric constant of the insulation layer and *t*_ins_ is the thickness of that layer. The areas *S* of a piezoelectric element and an insulation layer are the same because the piezoelectric film is deposited on the insulation layer. In this study, several investigations were conducted. First, to understand the influence of the parasitic capacitance, the thin film is segmented into elements with the same area. These elements are assumed to be subjected to the same stress (*i.e.*, generating the same initial voltages). Next, the series-connected elements with linear stress on a cantilever of a sensor are evaluated, and the number of connections and the locations are optimized.

### 3.1. Calculations for PZT Elements with Uniform Stress

First, we evaluated influential factors that affect the piezoelectric thin film segmented into equal areas, *S*/*n*, with uniform stress. The individual piezoelectric elements have the same initial voltages which are proportional to the piezoelectric constant. For simplification, the initial voltage is set to 1 V, and the resulting output voltage is evaluated as the multiplication factor. If the thin film is segmented into elements with stresses that are the same as that of the original thin film, the output voltage, *V*_n_, is equal to the multiplication factor, *M*, which is expressed as follows:

(3) $$M=\sum_{j}\sum_{k}K_{jk}$$

where **K** = (**I** + **GC_p_**^−1^**C**)^−1^.

**Figure 5.**
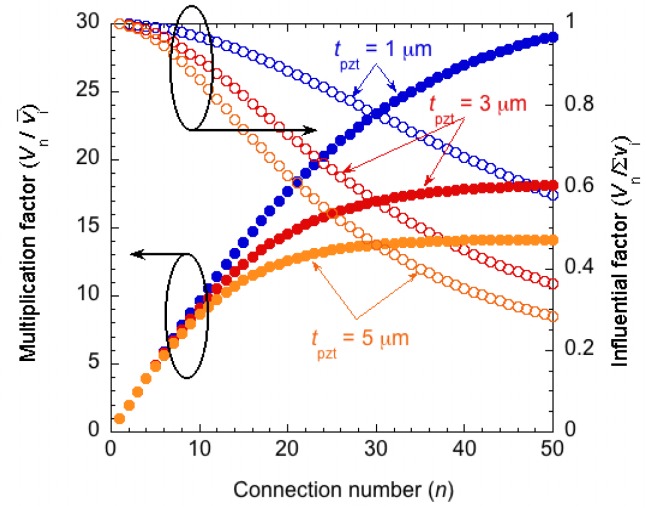
Calculated results using SPICE for series-connected PZT elements.

As an example, series-connected PZT elements (with a relative dielectric constant of 1,000 and an area of 1 mm^2^) placed on a SiO_2_ insulation layer (with a relative dielectric constant of 4 and a thickness of 1 μm) are evaluated for various numbers of segments. The initial area does not affect the relationship between the number of connections and the multiplication factor because the solution of Equation (2) can be expressed as the ratio of the capacitance of the piezoelectric elements to the parasitic capacitances. The thickness of the piezoelectric thin film is directly related to the capacitance of the piezoelectric elements. Figure 5 shows the result of the SPICE calculation for piezoelectric thin films with various thicknesses. The multiplication factor saturates when the number of connections increases. This saturation is due to the influence of the parasitic capacitances. The multiplication factor of the output voltage is limited to approximately 18 for a 3-μm-thick PZT even with 50 connections. Assuming an ideal, parasitic capacitance-free circuit, the output voltage should be proportional to the number of connections. The expected output voltage indicated by a series connection of PZT is ideally the simple summation of output voltages produced by all elements. The influential factor of the parasitic capacitance can be defined by the ratio of the obtained output voltage to the summation of the output voltage from all elements. For example, the influential factor is 0.5 for a 27-element connection with a thickness of 5 μm.

**Figure 6.**
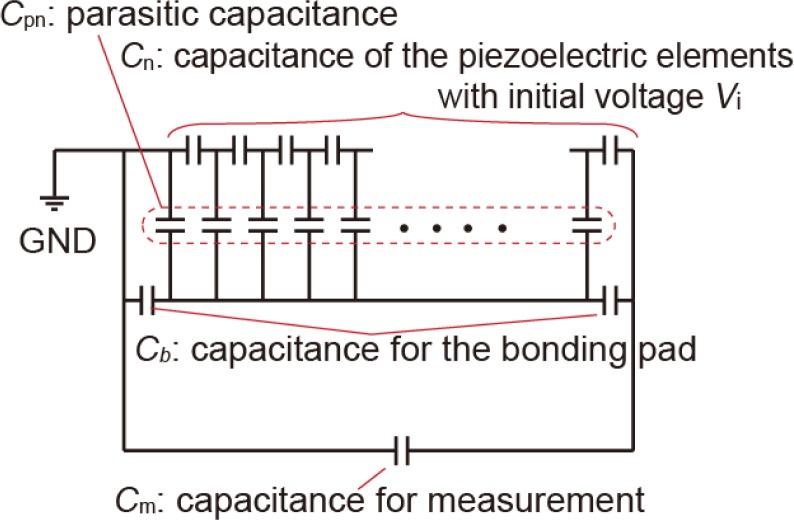
Equivalent-circuit model of series-connected piezoelectric elements. The piezoelectric elements are modeled as simple capacitors with initial voltages.

**Figure 7.**
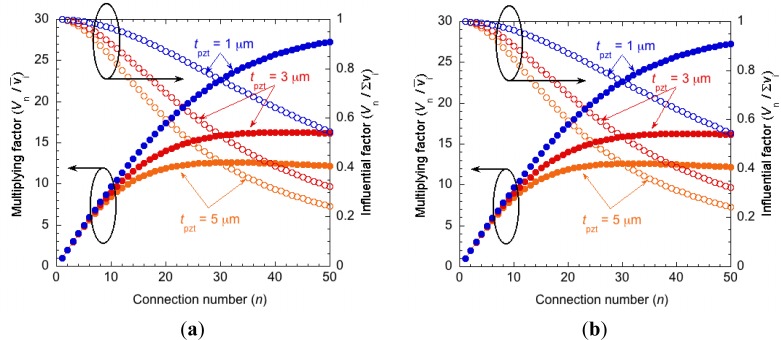
Influence of the parasitic capacitance. (**a**) Series-connected piezoelectric elements with bonding pads; (**b**) Series-connected piezoelectric elements with bonding pads and measurement instruments.

For actual systems, other parasitic capacitances are also present. For example, the insulation layer under bonding pads forms a parasitic capacitance, and measurement instruments also have parasitic elements. Figure 6 shows the equivalent circuits. The output voltage is calculated by considering parasitic capacitances derived from two bonding pads with an area of 150 × 150 μm^2^ and assuming a 10 pF probe capacitance. Figure 7(a,b) indicate the results of the calculations considering these parasitic capacitances. The output voltage is more restricted than in the case without bonding pads and measurement-derived parasitic capacitances. There is an optimum number of connections for the multiplication factor, depending on the thickness of the PZT. This optimum number is derived from the parasitic capacitance of the measurement circuit. If the device is directly connected to the peripheral circuit, there is no optimum number. Although these calculations are performed for PZT, the influence of the parasitic capacitance increases for piezoelectric materials with low dielectric constants such as AlN, ZnO, and poly-vinylidene fluoride.

### 3.2. Calculations for Series-Connected Piezoelectric Elements on a Cantilever

For MEMS devices, cantilever-type sensors are commonly used (e.g., accelerometers have proof mass with a supporting cantilever). We evaluated the influence of the parasitic capacitance for series-connected piezoelectric elements on a cantilever. When a horizontal cantilever with length *l*, width *w*, and thickness *t* bends in the vertical direction, segmentation in the width direction (an element has width *w*/*n* and length *l*) results in equal stress on the individual elements. Therefore, the output voltage can be estimated to be the same as that in Section 3.1. The segmentation in the length direction distributes the stress into the segmented elements. When a load *P* is applied onto the tip of a cantilever with length *l*, width *w*, thickness of the non-piezoelectric substrate *t*_s_, and piezoelectric element *t*_p_, the mean stress across the piezoelectric element σ(*x*) at a position *x* units away from the free edge on the cantilever is given as follows:

(4) $$\sigma \left(x\right)=\frac{E_{s}E_{p}t_{s}^{2}}{\left(E_{s}t_{s}^{3}+E_{p}t_{p}^{3}\right)\left(E_{s}t_{s}+E_{p}t_{p}\right)}\frac{6P}{w}x$$

**Figure 8.**
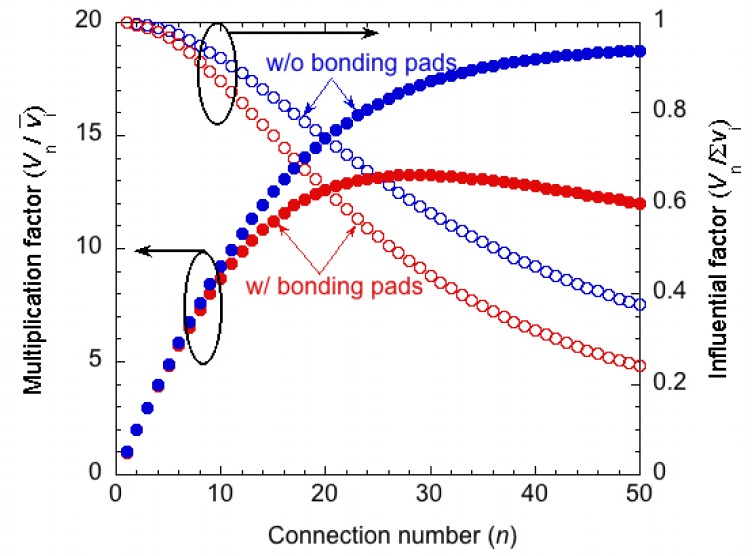
Influence of the parasitic capacitance for piezoelectric elements on a cantilever.

Therefore, the output voltage generated from the element is proportional to the distance from the free end of the cantilever. The calculation is performed using SPICE with the piezoelectric thin film (with dimensions of *l* = 1 mm, *w* = 0.1 mm, and *t*_p_ = 3 μm) segmented into *n* elements having the same length. In the SPICE calculation, the initial voltage on the *i*-th element is given as follows:

(5) $$V_{i}=\frac{V_{0}}{n}\left(i-\frac{1}{2}\right)$$

where *V*_0_ (set to 1 V for simplification) is the apparent voltage, which is estimated from the stress on the fixed end. Figure 8 shows the result. The multiplication factor is the ratio of the calculated output voltage to that without segmentation (0.5 V for the cantilever with the apparent initial voltage of 1 V at the fixed end). The multiplication factor decreases as the number of segments decreases, as is the case with uniform stress (Figure 5). From Equation (2), the multiplication factor for series-connected piezoelectric elements with the same area on a cantilever is expressed as follows:

(6) $$M=\frac{lVn}{\int_{0}^{l}\left(x/l\right)dx}=\frac{1}{2}\sum_{j}\sum_{k}\left(Q{'}_{jk}/C\right)=\sum_{k}K\cdot V$$

where **V** is the vector composed of {*V*_1_, *V*_2_, . . . *V*_n_} in Equation (5). The parasitic capacitances of the bonding pads are also investigated, as shown in Figure 8. These capacitances are found to be even more influential. The parasitic capacitance of the bonding pads and that of the peripheral measurement circuits should be properly estimated during the design stage.

### 3.3. Calculations for Series-Connected Piezoelectric Elements on a Both-Ends-Fixed Beam

**Figure 9.**
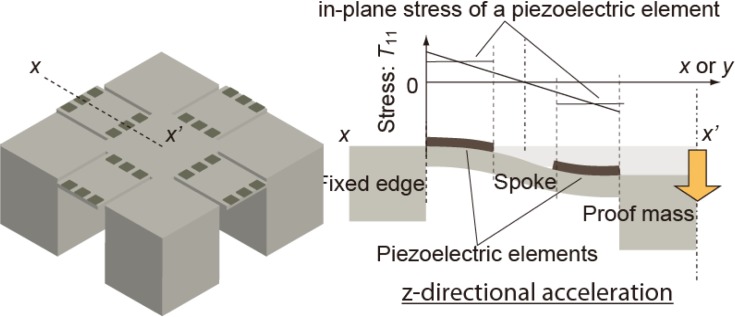
Tri-axis accelerometer using series-connected piezoelectric elements. The stress directions applied to the elements differ from each other.

In case of a tri-axis accelerometer, both-ends-fixed beams are often employed. For beams fixed at both ends, the sign of the stress (*i.e.*, compressive and tensile) is determined by location on the structure. For multiplication of the output voltage, the connection of the electrodes should be symmetrical as shown in Figure 1(b). Assuming a beam of a tri-axis accelerometer as in Figure 9, when a downward load *P* is applied to the proof mass, the stress in the piezoelectric thin film at distance *x* from the mass bearing on a beam with length *l* is expressed as follows:

(7) $$\sigma (x)=\frac{E_{s}E_{p}t_{s}^{2}}{\left(E_{s}t_{s}^{3}+E_{p}t_{p}^{3}\right)\left(E_{s}t_{s}+E_{p}t_{p}\right)}\frac{3P}{2w}(l-2x)$$

The beam center (*x* = *l*/2) is the border between the different stresses. When the piezoelectric elements are connected beyond the beam center, the elements should be connected symmetrically. With the symmetrical connection at the center, the output voltage of the connected piezoelectric elements is proportional to the distance *x*. Therefore, the influence of the parasitic capacitance has same result as that in Section 3.2. The output voltage decreases depending on the number of connections and parasitic capacitances derived from the bonding pads and measurement instruments. Note that in the equivalent circuits, some parasitic capacitances below the bottom electrodes for the symmetrical connections are connected to different positions than for the asymmetrical connections.

## 4. Discussion

While using output-voltage multiplication using a series connection of piezoelectric elements is a promising technique for piezoelectric MEMS sensors, it is important to estimate the influence of parasitic capacitances in the design stage. To investigate the influence of the parasitic capacitance, piezoelectric elements are modeled as a charge source with a capacitor. The influence of the parasitic capacitance is analytically expressed in Section 2.2. Although the analytical equations are useful to predict the influence of the parasitic capacitances, the model should be incorporated in SPICE for the extensibility, e.g., consideration of leakage and load resistance for energy harvesters. Since the charge source is not included in the standard SPICE library, the simplest model of a piezoelectric capacitance with initial voltage is proposed. In addition, the calculation using the SPICE model has been consistent with the experimental output voltage for the series-connected 8 piezoelectric elements in the previous work [14] (the focus has the multiplication of output voltage and the description of SPICE model in detail has not been indicated in the reference).

The demonstrated calculations indicate severe limitations on the output voltage because of the influence of parasitic capacitances. This effect depends on the ratio of the capacitance of the piezoelectric element to the parasitic capacitance. To reduce this influence, reduction of the ratio is one solution, *i.e.*, smaller parasitic capacitance and larger piezoelectric capacitance. For the small parasitic capacitance, the insulation layer making the parasitic capacitance should be thicker and have small permittivity. The additional parasitic capacitances such as insulators underneath bonding pads and the parasitic capacitance of peripheral circuits are also influential factors in the design of MEMS using the series-connected piezoelectric elements. Parasitic capacitances at the bonding pads can be reduced by minimizing the connection area between the output pads and peripheral circuits. The parasitic capacitance derived from the measurement system is critical and significantly limits the multiplication factor and optimum number of connections. Although the larger piezoelectric capacitance is effective to reduce the influence of the parasitic capacitance, enlargement in the permittivity of the piezoelectric material or thin piezoelectric film can result in the small electromechanical coupling coefficient and degrading the device performance. PZT has large electromechanical coupling coefficient and high permittivity. The high permittivity leads to large ratio of the piezoelectric capacitance to parasitic capacitance. Therefore PZT is suitable for the material of series-connected piezoelectric elements. The output voltages of series-connected piezoelectric elements with different stresses, such as that on a cantilever and a both-ends-fixed beam, are also analyzed. The output voltages are likewise decreased in the calculations for the series-connected piezoelectric elements with uniform stresses. The influence of the parasitic capacitance should be estimated for the devices utilizing series-connected piezoelectric elements.

The proposed technique using SPICE calculations can easily predict the influence of parasitic capacitance without the need for experiments. The areas and locations of the segmented elements for more complicated devices should be designed to maximize the output voltage based on the stress characteristics. In the design of actual devices, the piezoelectric elements should be connected in series by processing the electrodes and depositing the insulation layers. In addition, the fill factor of piezoelectric elements occupying the structure area, locations, and shapes used to load the stress are important. SPICE calculation in the design stage is a powerful tool for such piezoelectric devices.

## 5. Conclusions

The influence of the parasitic capacitance on series-connected piezoelectric elements is evaluated using the proposed SPICE calculations. We propose a simple-circuit model for piezoelectric elements to make it applicable to SPICE calculations. SPICE calculation easily estimates the influence of the parasitic capacitance on the output voltage of series-connected piezoelectric elements. The calculation results for series-connected PZT elements subjected to a uniform stress demonstrated the limitation of the output voltage based on the thickness of PZT. It was found that other parasitic capacitances derived from bonding pads and measurement instruments were also influential. By applying the technique to PZT elements located on beams, the design guidelines for series-connected piezoelectric devices have been explained.
